# Response of maize and common bean to spatial and temporal differentiation in maize-common bean intercropping

**DOI:** 10.1371/journal.pone.0257203

**Published:** 2021-10-01

**Authors:** Yayeh Bitew, Bitwoded Derebe, Abebe Worku, Gobezie Chakelie

**Affiliations:** 1 Bahir Dar University, Bahir Dar, Ethiopia; 2 Amhara Regional Agricultural Research Institute, Bahir Dar, Ethiopia; Anhui Agricultural University, CHINA

## Abstract

An experiment on maize (Zea mays)-common bean (*Phaseolus vulgaris* L.) intercropping was conducted for two years (2014 and 2016) at two locations in North western Ethiopia with the objective of determining the spatial arrangement and planting date of common bean. Common bean intercropped with maize at three planting dates (simultaneously with maize, at emergence and knee height of maize) in two spatial arrangements (alternate and paired arrangements).The experimental design was factotrial randomized complete block design with three replications. Sole maize and common bean were included as a check. Results revealed that the spatial and temporal differentiation significantly affect only the agronomic attributes of common bean in common bean-maize intercropping. At Adet the grain yield of common bean (1.9 t ha^-1^), LER (1.99) and MAI (357) in maize-common bean intercropping was higher when common bean was planted at the same time with maize in paired planting pattern. On the other hand, maximum LER (1.61) and MAI (2.83) at Finoteselam were observed when common bean was intercropped with maize at maize emergence in paired planting pattern. Simultaneous intercropping of common bean with maize gave more stable total land output yield as compared to other intercropping systems but showed high variability as compared to the sole cropping. Thus, it can be concluded that planting common bean simultaneously with maize in paired planting pattern at Adet and planting common bean at maize emergence at Finoteselam in maize-common bean intercropping gave maximum land use efficiency and profitability of the cropping system without reducing the main crop yield (maize).This research also suggested further research on the compatibility of various maize and common bean varieties in different spatial and temporal differentiation.

## Introduction

Over decades, food requirements in Ethiopia have increased geometrically while land availability has declined rapidly [1]. Agriculture had been characterized by a very low growth rate (1.4% annum^-1^), which was less than the growth rate of population (2.49% annum^-1^) [2]. So, the major challenge for researchers meeting here today is to assess the prospects for meeting food demand. Generally, there are two ways of increasing food production [1–3]: (i) horizontal growth by putting more land to agricultural production; (ii) vertical growth through increasing productivity per unite land. The first alternative is finite in scope and can go only a limited extent. A second alternative, on the other hand has more possibilities. Among these, intercropping of cereals with legumes is important for the development of sustainable food production [3].

Intercropping is the simultaneous cultivation of more than one crop species on the same piece of land and is regarded as the practical application of basic ecological principles such as diversity, competition and facilitation [4]. Some potential benefits of intercropping systems are increasing the productivity and profitability [5], improvement of soil fertility through nitrogen fixation by the component legume [6], efficient use of environmental resources [7], reducing damages caused by insect pests, diseases and weeds [8], and improvement of forage production and quality [9]. Most research findings showed that yield of intercropping is often higher than sole cropping [10–12]. This is mainly due to resources such as water, light and nutrients can be utilized more efficiently in intercropping than in sole cropping [13–15]. The underlying principle of efficient resource use in intercropping is that, if crops differ in the way they utilize environmental resources when grown together, they can complement each other and make better combined use of resources than when they are grown separately [16,17].

However, the success of intercropping systems is due to an enhanced temporal and spatial complementarity of resource capture, for which both aboveground and belowground parts of crops play an important role [18,19]. Therefore, intercropping seems relevant management options in improving the efficiency of this system. Maximum yield of the component crops in an intrcropping can be achieved by minimizing competition effects through appropriate planting pattern and timing of intercropping based on growth characteristics and requirements of the component species [8]. Even though, such agronomic options seem easily controllable management factors, their effects on intercrop yields need to be well understood and determined experimentally.

Morpho-physiological differences and agronomic factors such as the proportion of crops in the mixture regulate competition between component crops for growth-limiting factors [20]. The degree of yield loss due to competition in an intercropping depend on the competitive ability, planting density and relative planting time of the component crop species, planting arrangement and nutrient availability in the soil [19,20]. Thus, enhancing productivity of maize and bean intercrops requires improving the interspecies complementarity or reducing competition effects. This might be achieved through manipulation of plant arrangements, plant densities, relative planting dates and planting compatible cultivars [21,22].

Relay intercropping is one of the most important agronomic management decisions to consider when deciding to practice intercropping. It is a system in which a second crop is planted into an existing crop but before harvesting [23]. Relay intercropping is a good example of temporal complementarity of crop mixtures, in which there is an increase in the total leaf index because the second crop has built its seedling with relative larger leaf area index after the first cropis harvested [23–25]. Examples of relay crops are cassava, cotton, sweet potato and sesame with corn, chickpea, lentil,wheat with upland rice [23], grass pea with rice [24], maize with sunflower [25].

The other important management aspect is spatial arrangement which can improve radiation interception from complete ground cover and determine whether an intercrop system will be advantageous or not with regard to yield gains [26,27]. The highest LER (1.51) was obtained when the 50% population of maize was intercropped with the full population of sesame in the treatment where two rows of sesame were followed by two rows of maize [28]. Moreover, The highest number of seeds per pod and seed yeield was recorded with three rows of soybean to one row of ginger [29]. Maize-legume intercroppingis the dominant cropping system practiced by farmers in north western Ethiopia [30]. Most farmers intercropped legume crops at the same time with maize in alternate planting time without reducing the plant population of maize in order to achieve his/her major objective of food self-sufficiency. Various investigations in Ethiopia reported that most farmers have been practicing various intercropping systems such as maize-common intercropping,because of land scarcity and the need to reduce risk of crop failure caused by erratic rain fall, drought, pests etc. [31–33]. However, this practice was not scientifically improved yet. Thus, choice of appropriate plant population density in an intercropping using appropriate spatial arrangement; and determining of planting time of legume crops are the key management options in improving the efficiency of this producction system. Hence, the objective of this study was to determine the appropriate spatial arrangement and planting date of common bean in maize-common bean intercropping for maximum the production efficiency (Land use efficiency and crop equivalent yield) and the component yield in maize-common bean intercropping.

## Materials and methods

### Description of the study area

**Fig 1:**
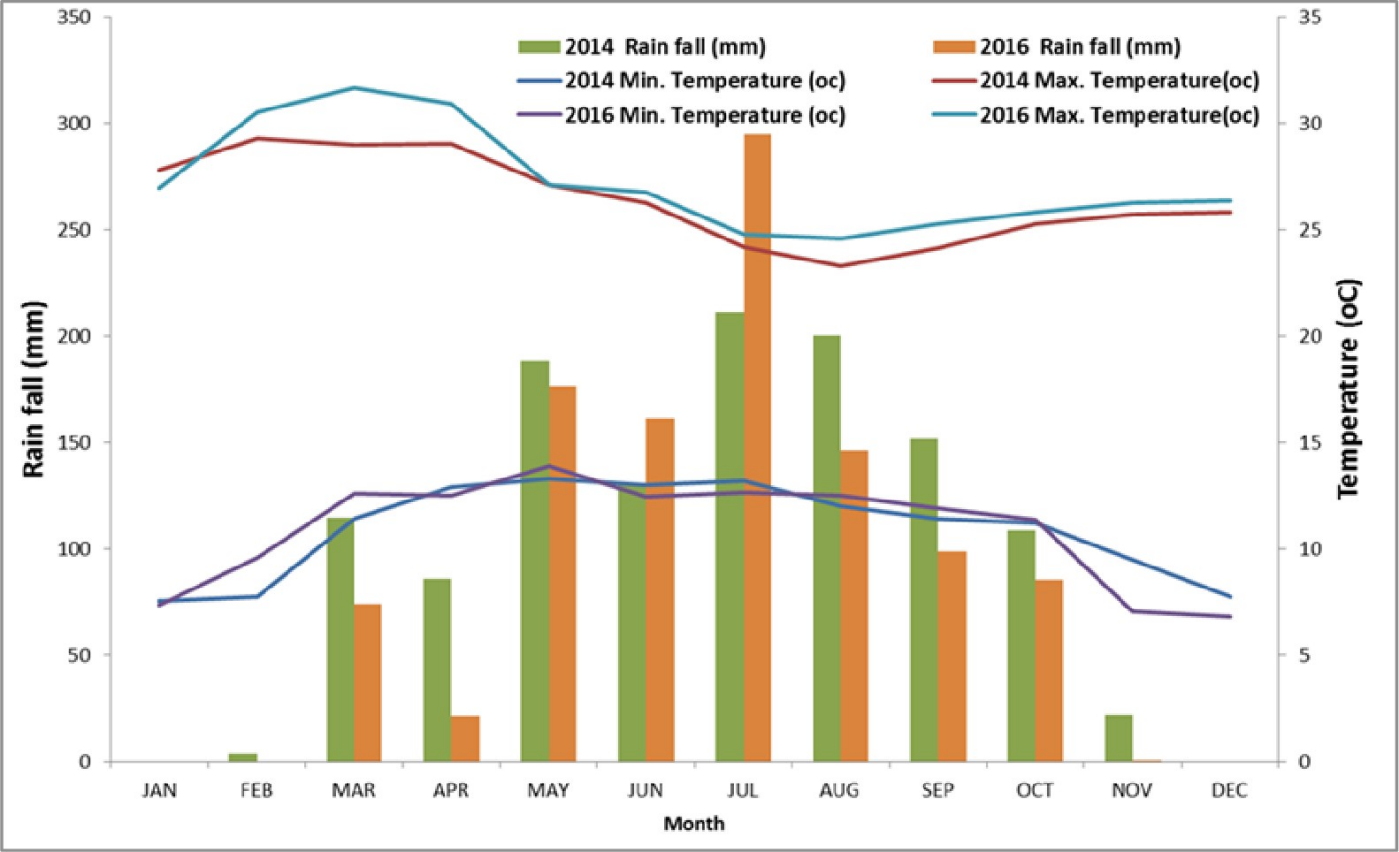
Mean monthly rainfall and temperature distribution in the two experimental years (2014 and 2016) at Adet.

**Fig 2:**
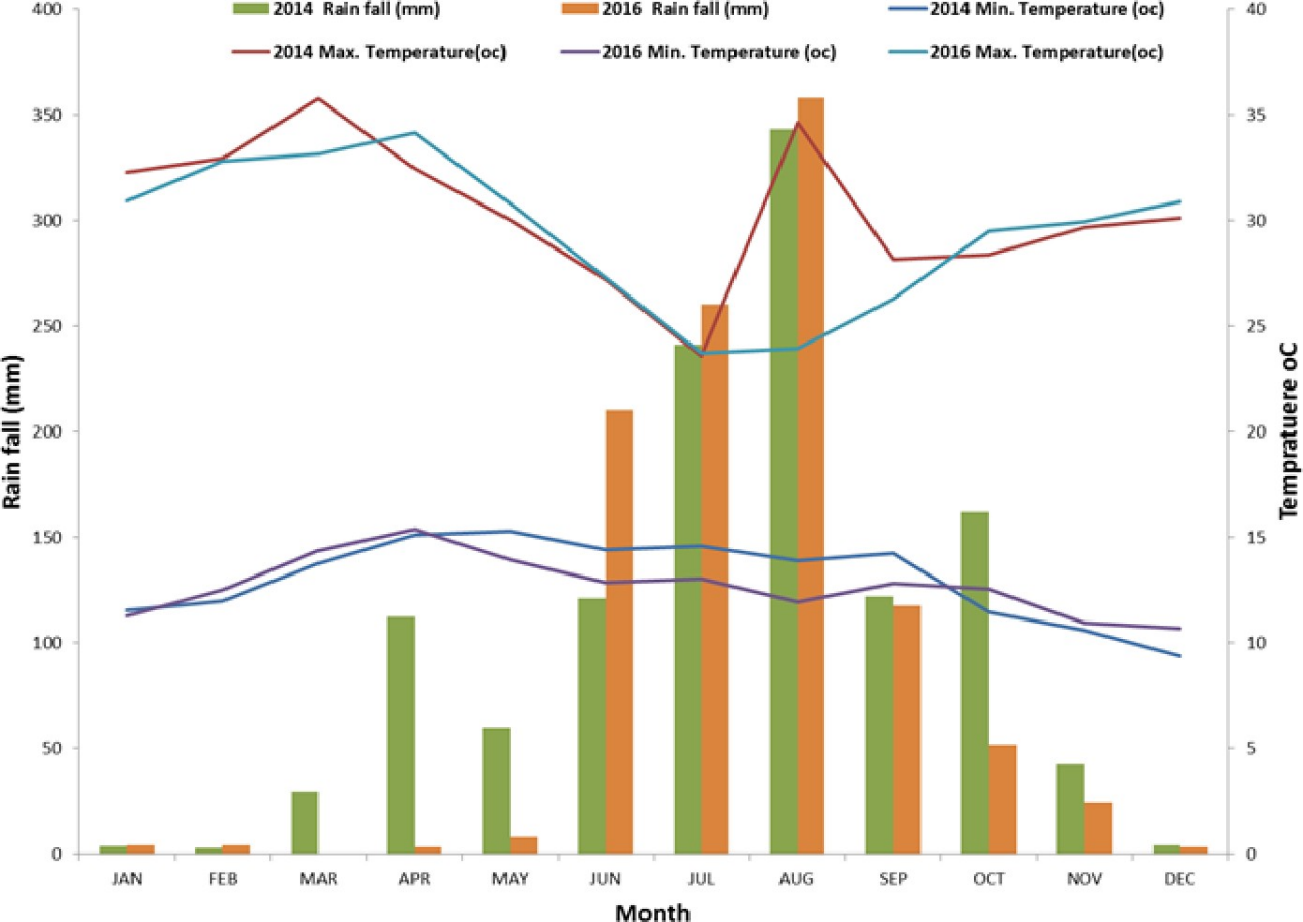
Mean monthly rainfall and temperature distribution in the two experimental years (2014 and 2016) at Finoteselam.

The experiment was conducted for two main rainy seasons (2014 and 2016) at Adet and Fenotselam Research Stations in North western Ethiopia. Adet research station is located between 11^0^17, N latitude and 37^0^ 43, E longitude with an altitude of 2240 meters above sea level (m.a.s.l.).Fenotselam research station is located between 37^0^ 16, E latitude and 10^0^ 42, N longitude with an altitude of 1917 m.a.s.l. At Adet the historical average mean annual precipitation is 1,372 mm andminimum and maximum temperatures are 11.0 and 26.8 ˚C, respectively [34]. At Finoteselam mean annual precipitation is 1272mm and the temperature typically varies from 11°C to 30°C [35]. At both locations the rainy season starts in June and ends in October [34,35]. Moreover, monthly weather data (rainfall and temperature) during the experimental years were obtained from the station office at Bahir Dar, Ethiopia, after it was collected in each experiemental locations [36].Thus, the total annual rainfall at Adet was 1215.20 mm and 1058.00 mm in 2014 and 2016, respectively (Fig 1).While at Finoteselam it was 1243.30mm and 1043.40mm in 2014 and 2016, respectively (Fig 2). The soil characteristics at Adet were 0.8%, 2.47%, 37.97 cmol (+) kg^-1^, 33.29 ppm, 1.98 ppm and 5.17 for total nitrogen, organic carbon, CEC, exchangeable K, available P and pH, respectively. Similarly, at Finoteslam the soil characteristics such as total nitrogen, organic carbon, available K, available P and pH were 0.02%, 3.57%, 6.164ppm, 1.98ppm and 5.17, respectively [35].

### Experimental treatments and design

Common bean was intercropped with maize at three planting dates of common bean (simultaneously with maize, at Emergence and knee height of maize) and two spatial arrangements (alternate and paired arrangements). Sole crops of maize and common bean were included to calculate the land use efficiency of the cropping systems. A total of eight treatments were laid out in a factorial randomized complete block design with three replications. Gross and net areas of experimental plots were 4.5m x 4.8m (21.6m^2^) and 2m x 4m (8m^2^). The distance between plots and replication were 0.5m and 1m, respectively.

### Experimental materials and procedures

**Fig 3:**
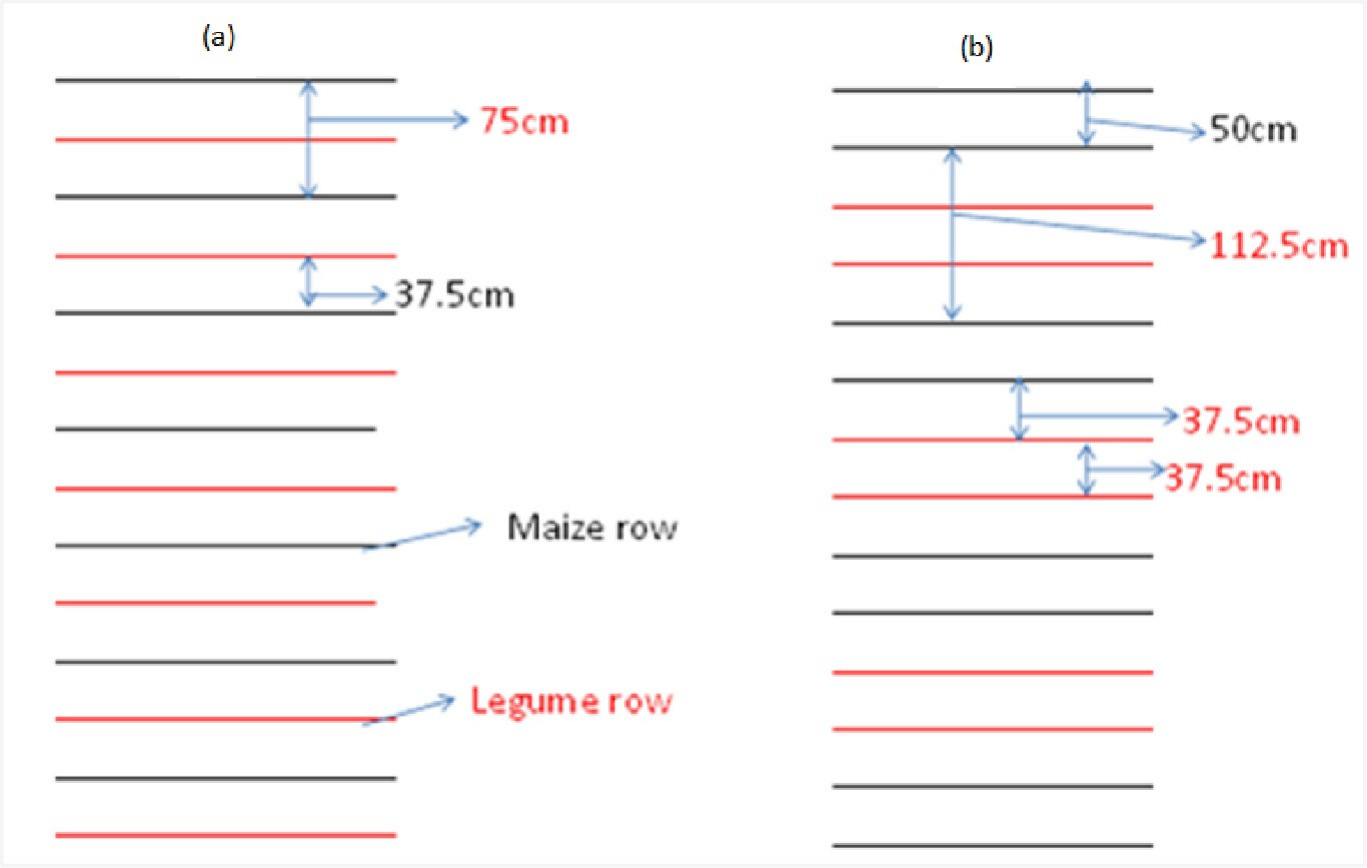
Spatial arrangement of common bean (a) alternate (b) paired in maize-common bean intercropping.

Hybrid maize variety ‘BH-540’ and common bean variety ‘Chorie’ were used as test crop for this experiment. The seeds were sourced from Adet Agricultural research center. The seed bed was tilled four times (passes) by using oxen plough following the local recommended crop cultivation package. In an alternate planting system (Fig 3A), maize was planted in 75cm x 25cm and common bean was planted between maize rows in 10cm between plants. In paired planting system, maize was planted in two row arrangements i.e., 50cm x 25cm and 112.5cm x 25cm (Fig 3B).Two rows of common bean were planted manually in 37.5cm x 10cm in the latter planting system. Sole maize and common bean were planted manually in 75cm x 25cm and 40cm x 10cm, respectively. Fertilizer rate of 180 kg ha^-1^ N and 138 kg ha^-1^ P_2_O_5_ in the form of UREA and DAP (Di- ammonium phosphate), respectively were applied in band for plots planted with maize. All phosphors and half nitrogen were applied at planting time, while the remaining half nitrogen was applied when maize hight was reached at knee height. For the sole common bean a nutrient rate of 18–46kg ha^-1^ N-P_2_O_5_ was applied in drill line at planting in the from of DAP. Generally, planting of maize was done at the end of May (Adet) and at beginning of June (Finoteselam). Other crop management practices were done following the locally recommended packages for each component crops.

### Data collection and measurements

#### Yield attribute and grain yield of the component crops

Plant height (cm) of the component crops was measured from ten randomly taken plants on the net plot at 90% physiological maturity. Number of pods plant^-1^ of common bean was determined from the ten sampled plants of a net plot at physiological maturity. Number of seeds pod^-1^ of common bean was further determined from 15 pods taken from the ten sampled plants. The above ground biomass of the component crops were measured after the plants from the net plot area were harvested and sun dried till constant dry weight attained. Similarly, grain yield of both componant crops was determined after the grain had been dried, threshed, cleaned and adjusted to their respective optimum moisture level. Grain moisture content of the component crops were measured using a grain moisture tester and their grain yields were adjusted to the moisture content of 12.5% for maize and and 10% for common bean.

#### Evaluation of production efficiency and profitability

*Land equivalent ratio (LER)*. In assessments of crop productivity of sole cropping systems, a useful expression is the mass yield (mass per unit area). However, in intercropping systems, direct comparison is difficult because products are different for the different plant species growing on one piece of land [37]. In this case, crop productivity should be evaluated using a common unit. A widely used method is the land equivalent ratio (LER) [37]. It indicates the efficiency of intercropping for using the resources of the environment compared with mono-cropping [38]. The key assumption in the use of the LER is that the densities of plants in the sole cropping controls are close to the optimum. The value of unity is the critical value. The null hypothesis (LER = 1) mean that inter- and intra- specific interactions are equivalent. When the LER is greater than one, the intercropping favors the growth and yield of the species. In contrast, when LER is lower than one the intercropping negatively affects the growth and yield of the plants grown in mixtures. The LER was calculated using the formula outlined by Mead and Willy [39]:

$$LER=\sum_{i=1}^{n}(\frac{Yi}{Ym})$$ (1)

Where, Yi and Ym are yields of component crops in intercrop and monulture system, respectively and n is the number of crops involved.

*Land equivalent coefficient (LEC)*. The land equivalent coefficient is calculated according to the formula stated by Ejigu *et al*. [40]:

$$LEC\;(\%)=\sum_{i=1}^{n}LER1*LER2$$ (2)

Where, LEC = land equivalent coefficient; LER1 = partial land equivalent ratio of crop 1 and LER2 = partial land equivalent ratio of crop 2. The inference is that in intercropping system, the minimum expected land equivalent coefficient is 25% and if LEC value exceeds 25%, there is the yield advantage [40].

*Crop equivalent yield (CEY)*. Production efficiency can also be assessed by CEY [41] as

$$CEY\;(kg\;ha-1)=\sum_{i=1}^{n}Ys\;(kg\;ha-1)\;(\frac{Ps(ETB\;kg-1)}{Pm(ETB\;kg-1)})$$ (3)

Where, CEY is crop equivalent yield; Ys is the yield of supplementary crops (kg ha^-1^), Ps (30 kg ha^-1^) and Pm (12 kg ha^-1^) are the average price of the supplementary and main crops, respectively in Ethiopian Birr per kg (ETB kg^-1^), during their production years. In this case the main and the supplementary crops were maize and common bean, respectively. To calculate the total crop equivalent yield, maize yield and crop equivalent yield were summed up and expressed as t ha^-1^.

*Monetary advantage index (MAI)*. Monetary advantage index (MAI) was calculated according to Hirpa [42]:

$$MAI=\sum_{i=1}^{n}M1+M2*\frac{LER-1}{LER}$$ (4)

Where, MAI = monetary advantage index; M1 = P1 ×Y1; M2 = P2 ×Y2; P1 = Price of crop 1 (maize) and the P2 = Price of crop 2 (common bean); the average price of maize and common bean seed kg^-1^ in Ethiopian Birr was taken from the local grain market during the cropping season. Accordingly, the prices were 6.5 and 20 Birr ETB (Ethiopian Birr) kg^-1^ for maize and common bean respectively. The higher the MAI value, the more profitable the cropping system is [43].

### Data and yield stability analysis

Data on yield attributes and grain yield of the component crops were analyzed following two-way analysis of variance (ANOVA), using JMP13 software [44] for each location and year. The data were analyzed separately for each location since values for the error mean square of the two sites were heterogonous [45]. In the analysis, year and replication were considered as a random variable and locations were considered as a fixed variable. When the analysis of variance showed significant differences among treatments in any level of probability, mean separation for all pairs were conducted using Tukey-Kramer HSD. Using the same software regression analysis was done to know the relationship between the component crop grain yields as affected by spatial arrangement and planting time of common bean in maize-common bean intercropping.

Yield stability in intercropping was evaluated by comparing the coefficient of variation (CV) of different cropping systems [17]. The CV was defined as the ratio of standard deviation and mean value of the yield in the cropping system at different years and locations multiplied by hundred. It was taken as the response variables and each cropping system as explanatory variables. A higher CV indicated lower yield stability and vice versa [46].

## Results and discussion

### Maize yield attribute and yield

At both experimental locations with the exception of stover yield at Finoteselam, the results of the experiment showed that all the yield and yield components of maize were not significantly (P>0.05) affected by spatial arrangement and planting date of common bean, and by their interaction effects (Tables 1 and 2). At Adet, plant height, grain yield and thousand seed weight of maize were ranged from 363cm to 382cm, 6 t ha^-1^6.84 t ha^-1^, 245 grams to 253gram, respectively, while at Finoteselam the respective parameters were ranged from 305cm to 328cm, 5.86 t ha^-1^ to 9.42 t ha^-1^ and 235 to 270 gram, respectively (Tables 1 and 2).The non-significant effect of the spatial arrangement and planting date of common bean and their interaction on the yield attribute and grain yield of maize could be due to (i) both component crops have large temporal niche differentiation as a result of low plant growth resource competition; (ii) the spatial arrangement of common bean might reduce the shading effect of the common bean thereby favoring more efficient photosynthesis. Thus, Optimized planting time of common bean reduce the yield difference between intercropped and sole maize by enhancing maize resilience toward asymmetric competition. Parellele to this finding Ahmed et al. [47] documented that Optimized co-growth duration of soybean in Soyabean-maize intercropping reduce the yield difference between intercropped and sole soybean. Moreover, authors have reported that the yield of main crops did not vary significantly with the staggered sowing of the intercrop [48–51]. On contrary, Addo-Quaye *et al*. [52] found that maize planted simultaneously with soybean or before soybean gave significantly higher values of leaf area index, crop growth rate and net assimilation rate. Besides, Isaac et al. [28] claimed that the number of cobs per plant, cob length, and circumference of maize were not significantly influenced by spatial arrangement in maize-seasome intercropping. However, the same author reported plant height, stem girth; number of grains per cob, weight of grains, and yield were significantly influenced by spatial arrangement.

**Table 1:** Response of maize yield attribute and yield to spatial arrangement and planting time of common bean at Adet research station, Northwestern Ethiopia^a^.

Source of variation and statistics		PH (cm)	SY(t ha^-1^)	TSW (g)	GY(t ha^-1^)
PDCB	SA				
**Simultaneously**	Alternate	374.30	7.93	251.40	6.56
**At emergence**		382.17	9.35	245.40	6.84
**At knee height**		369.77	7.57	248.73	6.67
**Simultaneously**	Paired	367.70	7.67	252.13	6.45
**At emergence**		363.83	7.76	250.53	6.06
**At knee height**		374.50	7.53	252.87	6.36
**Mean**		372.05	7.97	250.18	6.49
**LSD (5%)**		Ns	Ns	Ns	ns
**CV (%)**		1.76	9.50	4.80	8.40
**SA**		Ns	Ns	Ns	ns
**PT**		Ns	Ns	Ns	ns

^a^Data was combined over years (2014 and 2016). PDCB = Planting date of common bean with maize in a mixture; SA = spatial arrangements of the component crops; PH = plant height (cm); SY = stover yield (t ha^-1^); TSW = thousand seed weight (gram); GY = grain yield (t ha^-1^); SA = spatial arrangement; PT = planting time of common bean; ns is non-significant at 0.05 probability level.

**Table 2:** Response of maize yield attribute and yield to spatial arrangement and planting time of common bean at Finoteselam sub-research station, Northwestern Ethiopia^a^.

Source of variation and statistics		PH (cm)	SY (t ha^-1^)	TSW (gram)	GY (t ha^-1^)
PDCB	SA				
**Simultaneously**	Alternate	323.50	9.79^c^	252.63	5.86
**At emergence**		321.50	12.53^b^	256.85	7.68
**At knee height**		314.17	14.32^a^	269.76	8.67
**Simultaneously**	Paired	305.33	7.80^d^	235.64	6.92
**At emergence**		314.83	11.64^bc^	269.10	9.42
**At knee height**		328.00	11.30^bc^	262.06	9.17
**Mean**		317.89	11.23	257.67	7.95
**LSD (5%)**		Ns	**	Ns	Ns
**CV (%)**		4.30	14.60	5.40	17.90
**SA**		Ns	Ns	Ns	Ns
**PT**		Ns	*	Ns	Ns

^a^Data was combined over years (2014 and 2016). PDCB = Planting date of common bean with maize in a mixture; SA = spatial arrangements of the component crops; PH = plant height (cm); SY = stover yield (t ha^-1^); TSW = thousand seed weight (gram); GY = grain yield (t ha^-1^); SA = spatial arrangement; PT = planting time of common bean

** is significant 0.01 probability level; Ns is non-significant at 0.05 probability level.

### Common bean yield attribute and yield

At Adet results showed that agronomic parameters (plant height, seeds pod^-1^, pod plant^-1^, biomass yield, grain yield and thousand seed weight) were significantly (P<0.05) affected by the interaction between spatial arrangement and planting time of common bean (Table 3). At Finoteselam, except pod plant^-1^, plant height, seeds pod^-1^, biomass yield, grain yield and thousand seed weight were significantly (P<0.05) affected by the interaction between spatial arrangement and planting time of common bean (Table 4).The highest common bean plant height at Adet and Finoteselam was observed when common bean and maize was planted simultaneously in alternate (94cm) and paired (89cm) planting pattern, respectively (Tables 3 and 4). The highest common bean seed pod^-1^ at Adet was observed when common bean and maize was planted simultaneously in paired planting pattern (7.17) (Table 3) while at Finoteselam it was obtained when common bean was planted at knee height of maize in paired planting pattern (8.67) (Table 4). At both locations the highest biomass yield was recorded when common bean was planted at same time with maize in paired and alternate planting pattern. At Finoteselam and Adet, the highest grain yield was recorded when common bean was planted at same time with maize in alternate and paired planting pattern, respectively (Table 3 and 4). Similar to this result, Alemayehu et al. [53] indicated that simultaneous maize and common bean planting gave the highest common bean grain yield as compared to subsequent plantings.

**Table 3:** Response of common bean yield attribute and yield to its planting time and spatial arrangement in maize -common bean intercrops at Adet research station, Northwestern Ethiopia ^a^.

Source of variation and statistics		PH (cm)	PPP	SPP	SY (t ha^-1^)	GY (t ha^-1^)	TSW (gram)
PDCB	SA						
**Simultaneously**	Alternate	94.00^a^	16.77^ab^	6.50^a^	3.97^a^	0.99^b^	258.13^a^
**At emergence**		79.57^ab^	19.80^ab^	5.90^a^	2.36^b^	0.61^b^	266.83^a^
**At knee height**		15.97^c^	1.97^c^	2.03^b^	0.26^c^	0.01^c^	71.83^b^
**Simultaneously**	Paired	89.20^b^	25.00^a^	7.17^a^	4.16^a^	1.49^a^	263.87^a^
**At emergence**		77.47^ab^	22.07^ab^	6.87^b^	2.37^b^	0.72^b^	266.73^a^
**At knee height**		12.47^c^	2.50^c^	2.00^b^	0.24^c^	0.01^c^	71.50^b^
**Mean**		61.45	14.69	5.08	2.23	0.64	199.82
**LSD (5%)**		***	**	**	*	**	**
**CV (%)**		9.50		13.20		23.60	5.20
**SA**		Ns	*	Ns	Ns	*	Ns
**PT**		**	*		**	**	**

^a^Data were combined over years (2014 and 2016). PH = plant height; PPP = pod per plant; SPP = seed per pod; SY = stover yield; GY = grain yield; TSW = thousand seed weight; SA = spatial arrangement; PT = planting time

*, **’*** are significant at 0.05, 0.01, 0.001 probability level, respectively and Ns is non-significant at 0.05 probability level.

**Table 4:** Response of common bean yield attribute and yield to its planting time and spatial arrangement in maize -common bean intercrops at Finoteselam sub-research station, Northwestern Ethiopia ^a^.

Source of variation and statistics		PH (cm)	PPP	SPP	SY (t ha^-1^)	GY (t ha^-1^)	TSW (gram)
PDCB	SA						
**Simultaneously**	Alternate	46.07^b^	21.43	7.63^ab^	2.74^a^	1.02^a^	278.85^ab^
**At emergence**		59.00^a^	19.20	7.80^ab^	1.83^a^	0.55^bc^	269.68^abc^
**At knee height**		32.80^c^	12.53	7.17^b^	0.82^b^	0.35^c^	236.92^bc^
**Simultaneously**	Paired	56.37^ab^	18.87	7.83^ab^	2.55^a^	0.85^ab^	275.40^a^
**At emergence**		59.07^a^	19.13	7.73^ab^	1.88^a^	0.67^abc^	272.13^ab^
**At knee height**		35.07^c^	13.53	8.67^a^	0.81^b^	0.34^c^	230.02^c^
**Mean**		48.06	17.45	7.81	1761.65	629.89	260.50
**LSD (5%)**		*	Ns	*	*	*	*
**CV (%)**		8.2	19.7	6.2	26.8	20.7	5.6
**SA**		*	Ns	*	ns	Ns	ns
**PT**		**	**	Ns	**	**	**

^a^Data were combined over years (2014 and 2016). PDCB, Planting date of common bean with maize in a mixture; SA, spatial arrangements of the component crops; PH = plant height; PPP = pod per plant; SPP = seed per pod; SY = stover yield; GY = grain yield; TSW = thousand seed weight; SA = spatial arrangement; PT = planting time

*, **’*** are significant at 0.05, 0.01, 0.001 probability level, respectively and ns is non-significant at 0.05 probability level.

**Fig 4:**
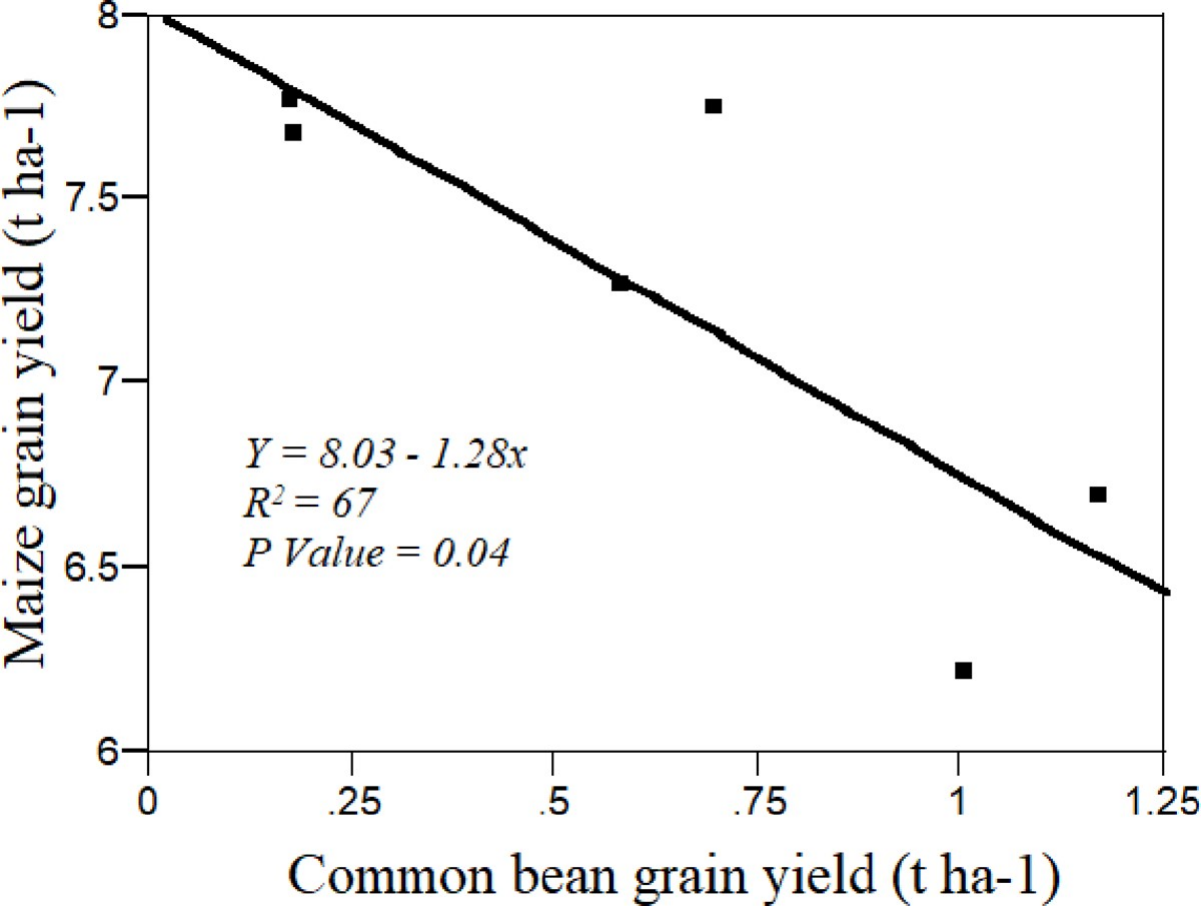
The relationship between the component grain yields as affected by spatial arrangement and planting date of common bean in maize-common bean intercropping.

Generally, the highest common bean yield attributes and yield were observed when it was planted with maize at the same time followed by at maize emergence in both alternate and paired planting arrangement compared to other traetements. On the average, at both locations the agronomic attributes of common bean was reduced as its planting time extended along the cropping seasons. Regression analysis also showed that there was an indirect relationship between the component grain yield as affected by spatial arrangement and planting date of common bean (Fig 4).The most important factor that caused the reduction of these common bean agronomic attributes as it was planted (intercropped) late in maize-common bean intercropping system might be due to (i) high amount of moisture from rainfall at the late seasons might affect the growth and N-fixation of common bean; (ii) the growth of common bean might be affected by shading at the late stage of maize. Parallel to this result, numerous researches on legume-non legume intercropping system showed that earlier sown component crops (legumes) showed higher growth and yield than late sown in an intercropping [54–57]. Similarly, Yayeh and Fekremariam [24] reported that the grain yield of chickpea was completely failed in rice pea relay intercropping in long temporal separation compared with short temporal separation.

### Land use efficiency and monetary advantage index

Results showed that partial land equivalent ratio (PLER) of both component crops were less than one for all treatments in both locations (Table 5). In both locations, all the partial LER of maize was higher than 0.5, while, in most cases, partial LER of common bean were lower than 0.5 which indicates that there was an advantage for maize and a disadvantage for common bean in these intercropping systems. This was in agreement with the works of Chen *et al*. [58], who reported that partial LER for cowpea was lower than 0.5 which indicated advantage for cotton in intercropping. While, partial LER for sorghum was more than 0.5, it indicated disadvantage for cotton in intercropping.

**Table 5:** Partial and total land equivalent ratios for maize-common bean intercropping at Adet research station, Northwestern Ethiopia^a^.

Treatments		Grain yield		Land use efficiency				MAI
PDCB	SA	Maize	Common bean	PLERM	PLERCB	LER	LEC (%)	
**Simultaneously**	Alternate	6.56	0.99	0.81	0.79	1.60	63.99	234.15
**At emergence**		6.84	0.61	0.84	0.49	1.33	41.16	140.58
**At knee height**		6.67	0.01	0.82	0.01	0.83	0.82	-89.21
**Simultaneously**	Paired	6.45	1.49	0.79	1.19	1.99	94.01	356.82
**At emergence**		6.06	0.72	0.75	0.58	1.32	43.50	130.40
**At knee height**		6.36	0.01	0.78	0.01	0.79	0.78	-110.42
**Sole maize**		5.31	-	1.00	-	1.00	-	0.00
**Sole common bean**		-	0.78	-	1.00	1.00	-	0.00

^a^Data were combined over years (2014 and 2016). PDCB, Planting date of common bean with maize in a mixture; SA, spatial arrangements of the component crops; PLERM = partial land equivalent ratio of maize; PLERCB = partial land equivalent ratio of common bean; LER = land equivalent ratio; LEC = land equivalent coefficient; MAI = monetary advantage index.

**Fig 5:**
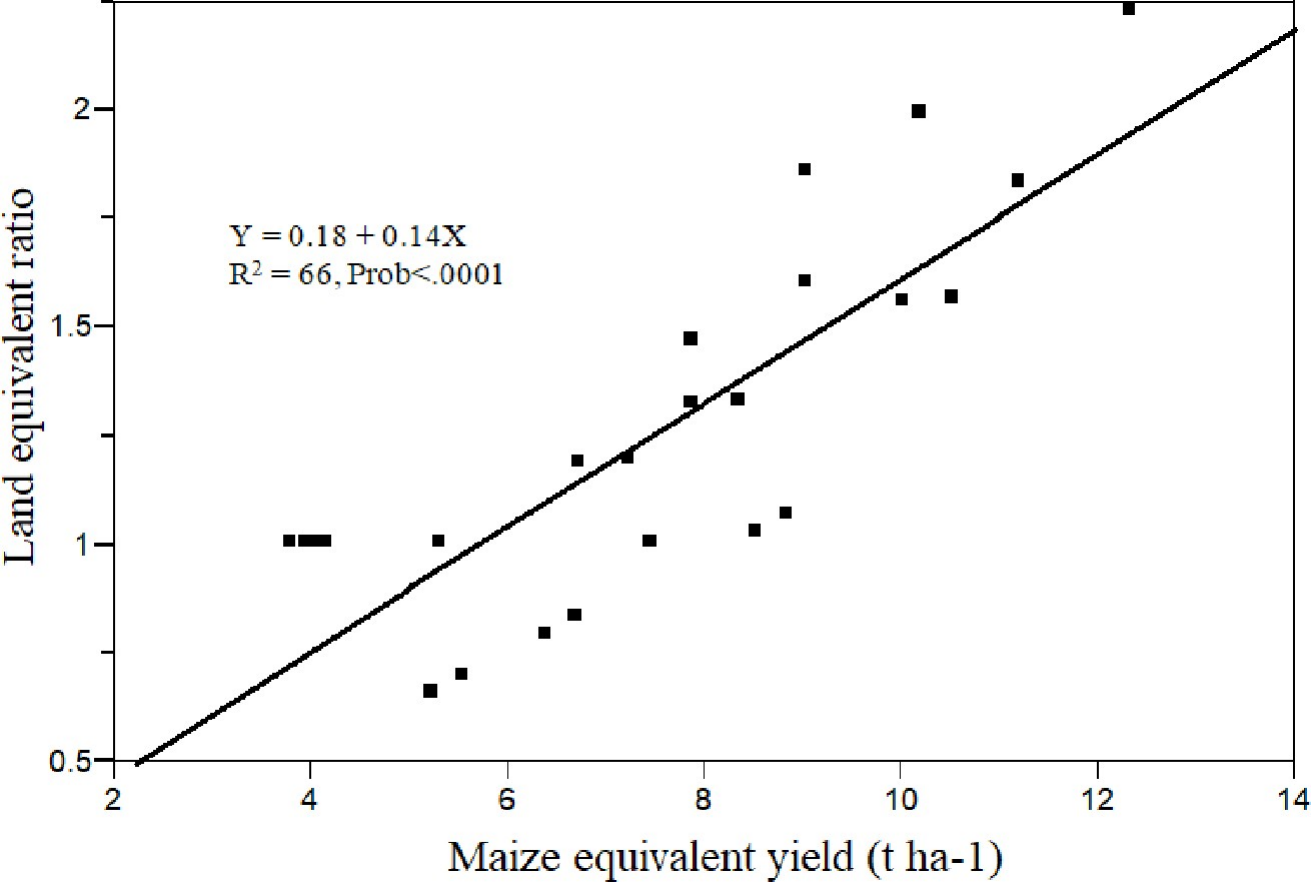
The relationship between the MEY and LER as affected by spatial arrangement and planting date of common bean inmaize-common bean intercropping.

At both locations in all treatments except common bean planted at knee height of maize in both planting pattern, LER was greater than one, which shows an advantage of intercropping and disadvantage of pure stands in terms of the use of environmental resources for plant growth. This is further explained by the direct relationship between LER and maize equqivalent yield (MEY) (Fig 5). Among the treatments, the yield advantage in terms of LER at Adet was greatest when component crops are planted at the same time in paired (1.99) followed by alternate (1.60) planting pattern. This indicates that using the respective intercropping systems 0.99 ha (99%) and 0.60 ha (60%) more area would be required by a sole cropping system to equal the yield of the respective intercropping systems. Likewise, at Finoteselam, the yield advantage in terms of LER was greatest when common bean was planted at emergence of maize in paired planting pattern (1.61) followed by when component crops were planted simoltanously in alternate (1.56) and paired (1.51) planting pattern. On an average, higher LER at both locations in both alternate and paired planting pattern was observed when common bean was planted at the same time with maize followed by planted at maize emergencee which might be due to an increase in grain yield of common bean as it was planted early in the main cropping season. Like LER, except planting of common bean at maize knee height in both planting pattern at Adet the land equivalent coefficient (LEC) was greater than 25%, indicating that these intercropping systems were a advantageous compared with sole cropping. Ashenafi [59], Belstie *et al*. [60] and Ejigu *et al*. [40] similarly reported LEC values greater than the critical in their study.

In intercropping systems, profitability to the growers is important [61] as overall success and adoptability of any system depends upon its economic feasibility. In this study, in all intercropping systems, except planting of common bean at maize knee height in both planting pattern at Adet, the MAI values were positive, which showed a definite yield advantage compared with sole cropping (Tables 5 and 6). Parallel to the values of all LEC, the highest positive MAI values at Adet were observed when common bean was planted at the same time in paired (356.82) followed by alternate (234.15) planting pattern. At Finoteselam, the highest positive MAI values were observed when common bean was planted at knee height of maize in paired arrangement followed by at same planting time in both alternate and paired planting arrangements. This indicated that these intercropping systems had the highest yield and economic advantage and implying the general suitability of common bean as an intercrop in maize production system. At both locations, the minimum positive MAI value was obtained when common bean was planted at knee height in both planting pattern (Tables 5 and 6). A plausible explanation for high MAI for the former cropping systems at both locations might be better utilization of growth resources between maize and common bean combinations and higher LER (Tables 5 and 6). Similarly, Ghosh [62] found that when the LER were higher there is significant economic benefit expressed with higher MAI. Similar findings were found in Pearl millet-cluster beans intercrop [63], wheat-rape intercrop [64], Vetch-barely intercropping [65], finger millet-lupine intercrops [33].

**Table 6:** Partial and total land equivalent ratios for maize-common bean intercropping at Finoteselam sub-resaerch station, Northwestern Ethiopia^a^.

Treatments		Grain yield (t ha^-1^)		Land use efficiency				MAI
PDCB	SA	Maize	Common bean	PLERM	PLERCB	LER	LEC (%)	
**Simultaneously**	Alternate	5.86	1.02	0.61	0.94	1.56	57.34	209.96
**At emergence**		7.68	0.55	0.81	0.51	1.31	41.31	144.16
**At knee height**		8.67	0.35	0.91	0.32	1.23	29.12	118.47
**Simultaneously**	Paired	6.92	0.85	0.73	0.79	1.51	57.67	209.34
**At emergence**		9.42	0.67	0.99	0.62	1.61	61.38	282.76
**At knee height**		9.17	0.34	0.96	0.31	1.28	29.76	145.26
**Sole maize**		3.57	-	1.00	-	1.00	-	0.00
**Sole common bean**		-	0.96	-	1.00	1.00	-	0.00

^a^Data was combined over years (2014 and 2016). PDCB, Planting date of common bean with maize in a mixture; SA, spatial arrangements of the component crops PLERM = partial land equivalent ratio of maize; PLERCB = partial land equivalent ratio of common bean; LER = land equivalent ratio; LEC = land equivalent coefficient; MAI = monetary advantage index.

### Yield stability

Generally, the coefficient of variations (CV) of the total land output yield (TLOY) was much higher than the individual crops in intercrops (Table 7). This result is contrary to the findings of Rao *et al*.[22], Vandermeer [17] and Yayeh *et al*. [19] who conducted a research in various cereal-legume intercrops. The CV of the component sole crops were lower than the component crops in an intercropping, indicating that the yield produced in sole cropping was more stable than in intercropping and this in line with farmers practices that cultivated sole cropping more than intercropping. The higher CV of component crops yield in intercropping than in a sole cropping could be due to higher interspecific competition between the component crops. Consistent with this result, Yayeh *et al*. [19] reported that intercropping of each sweet lupine and haricot bean with finger millet showed greater variability than sole crops. The CV of maize was 71% higher than common bean when the latter was planted with maize simultaneously and at emergence of maize in an alternate planting pattern (Table 7). On the remaining intercropping treatments, the CV of common bean was much higher than maize (Table 7). This might be due to temporal niche differentiation resulting in difference in stability of crops in various planting practices [19,22,66].

**Table 7:** Coefficient of Variation (CV %) for maize, common bean grain yield and TLOY in maize-common bean intercropping in north western Ethiopia^a^.

Intercropping treatments		Maize		Common bean		TLOY	
**PDCB**	SA	MGY (t ha^-1^)	CV %	MGY (t ha^-1^)	CV %	MGY (t ha^-1^)	CV %
**Simultaneously**	Alternate	6.21	7.97	1.01	2.11	6.21	102.02
**At emergence**		7.26	8.18	0.58	7.31	7.26	120.50
**At knee height**		7.67	18.44	0.18	133.56	7.67	134.94
**Simultaneously**	Paired	6.69	4.97	1.17	38.68	6.69	99.29
**At emergence**		7.74	30.70	0.70	5.09	7.74	118.12
**At knee height**		7.77	25.59	0.18	133.34	7.77	135.19
**Sole maize**		4.44	2.33	-	-	4.44	2.33
**Sole common bean**		-	-	0.87	2.25	0.87	2.25

^a^Data on maize and common bean and TLOY (MGY and CV) were combined over sites and years.

PDCB, Planting date of common bean with maize in a mixture; SA, spatial arrangements of the component crops; MG, mean grain yield; CV, coefficient of variation; TLOY, total land output yield.

## Conclusion

Results revealed that the spatial and temporal differentiation significantly affected only the agronomic attributes of common bean in common bean-maize intercropping. At Adet the grain yield of common bean, LER and MAI in maize-common bean intercropping was higher as common bean was planted at the same time with maize in paired planting pattern. At Finoteselam the grain yield of common bean was higher when common bean was intercropped with maize at the same time with maize in alternate planting pattern. However, maximum LER and MAI at Finoteselam were observed when common bean was intercropped with maize at maize emergence in paired planting pattern.Simultaneous intercropping of common bean with maize gave more stable total land output yield as compared to other intercropping systems but showed high variability as compared to the sole croppings. Thus, it can be concluded that planting common bean simultaneously with maize in paired planting pattern at Adet and planting common bean at maize emergence at Finoteselam in maize-common bean intercropping gave maximum land use efficiency and profitability without reducing the main crop yield (maize).This research also suggested further research on the compatibility of various maize and common bean varieties in different spatial and temporal differentiation.

## Supporting information

S1 Table Maize row data ready for analysis at Adet. (DOCX)

S2 Table Common bean row data ready for analysis at Adet. (DOCX)

S3 Table Land equivalent ratio row data ready for analysis at Adet. (DOCX)

S4 Table Maize row data ready for analysis at Finotslam. (DOCX)

S5 Table Common bean row data ready for analysis at Finotslam. (DOCX)

S6 Table Land equivalent ratio row data ready for analysis at Finotslam. (DOCX)

## References

[1] Derek H, Mekdim D, Alemayehu ST. Land constraints and agricultural intensification in Ethiopia: A village-level analysis of high-potential areas. Food Policy. 2014; 48:129–141.

[2] Gashaw TA, Zewdu BA, and Assefa AB. Effects of Land Fragmentation on Productivity in Northwestern Ethiopia. Advances in Agriculture. 2017; 1–9.

[3] Adesogan AT, Salawu MB, DeavilleE R. The effect on voluntary feed intake, invivo digestibility and nitrogen balance in sheep of feed-ing grass silage or pea- wheat intercrops differingin pea to wheat ra-tio and maturity. Anim. Feed. Sci. Technol. 2002; 96: 161–173.

[4] Hauggaard-Nielsen H, Bjarne J, Julia K, and Erik SJ. Grain legume–cereal intercropping: The practical application of diversity, competition and facilitation in arable and organic cropping systems. Renewable Agriculture and Food Systems. 2007; 23(1); 3–12.

[5] Yildirim E and Guvenc I. Intercropping based on cauliflower: More productive, profitable and highly sustainable. European Journal of Agronomy. 2005; 22(1):11–18.

[6] Hauggaard-Nieson H, Ambus P Jensen ES. Temporal and spa-tial distribution of roots and competition for nitrogen in pea-barley intercrops. A field studies employing 23 P techniques. Plant. Soil. 2001; 236: 63–74.

[7] Knudsen MT, Hauggaard-Nielsen H, Jornsgard B, and Jensen ES. Comparison of interspecific competition and N use in pea-barley, faba bean-barley and lupin-barley intercrops grown at two temperate locations. Journal of Agricultural Science. 2004; 142:617–627.

[8] Banik P, Midya A, Sarkar BK, Ghose SS. Wheatand chickpea intercropping systems in an additive experiment. Advantages andweed smothering. Europ. J. Agron. 2006; 24: 325–332.

[9] Bingol N, Karsli T, Yilmaz IH, Bolat D. The effects of planting time and combination on the nutrient composition and digestible dry mat-ter yield of four mixtures of vetch varieties intercropped with barley. J.Vet. Animal. Sci. 2007; 31: 297–302.

[10] Zhang F and Li L. Using competitive and facilitative interactions in intercropping systems enhances crop productivity and nutrient-use efficiency. Plant and Soil 2003; 248:305–312.

[11] Berntsen J, Hauggaard-Nielsen H, Olesen JE, Petersen BM, Jensen ES and Thomsen A 2004. Modeling dry matter production and resource use in intercrops of pea and barley. Field Crop Research. 2004; 88: 69–83.

[12] Dahmardeh M, Ghanbari A, Syasar BS, Ramroudi M. Effect of intercropping maize with cowpea on green forage yield and quality evaluation. Asian. J. Plant Sci. 2009; 8(3): 235–239.

[13] Dapaah HK, Asafu-Agyei JN, Ennin S A and Yamoah C. Yield stability of cassava, maize, soya bean and cowpea intercrops.The Journal of Agricultural Science. 2003; 140 (01): 73–82.

[14] Liu JH, Zeng ZH, Jiao LX, Hu YJ, Wang Y, Li H. Intercropping of different silage maize cultivars and alfalfa. Acta Agron. 2006; 32: 125–130.

[15] Malezieux E, Crozat Y and Dupraz C. Mixing plant species in cropping systems: concepts, tools and models. Agronomy for Sustainable Development. 2009; 29: 43–62.

[16] Ghanbari-Bonjar A. Intercropped wheat and bean as a low-input forage. PhD thesis. Wye College. Univ. London, 2000.

[17] Vandermeer J. The Ecology of intercropping. Cambridge Univ. Press, Cambridge, UK, 1989; p. 237. doi: 10.1016/0749-8063(89)90004-2

[18] Wu KX, Fullen MA, An TX, Fan ZW, Zhou F, Xue GF. Above- and below-ground interspecific interaction in intercropped maize and potato: A field study using the ’target’ technique. Field Crops Research 2012; 139: 63–70.

[19] Yayeh B, Getachew A, Enyew A & Alemayehu A. Competition, production efficiency and yield stability of finger millet and legume additive design intercropping. Renewable Agriculture & Food Systems. 2020; 36(1):1–12. doi: 10.1017/S1742170520000101

[20] Morgado L, Willey RW. Effect of plant population and nitrogen fertilizer on yield and efficiency of maize-bean intercropping. Sci. Agric. 2003; 38: 1257–1264.

[21] Mutungamiri A, Mariga IK and Chivinge OA. Effect of maize density, bean cultivar and bean spatial arrangement on intercrop Performance. African Crop Science Journal. 2001; 9(3): 487–497.

[22] Rao NG, Rana BS and Tarhalkar PP. Stability, productivity, and profitability of some intercropping systems in dryland agriculture. In Rana BS and Tarhalkar PP (eds), Proceedings of the International Workshop on Intercropping. Hyderabad, India, 1981; 10–13 January 1979: pp. 292–298.

[23] Lithourgidis AS, Dordas CA, Damalas CA and Vlachostergios DN. Annual intercrops: an alternative pathway for sustainable agriculture. Australian Journal of Crop Science. 2011; 5(4): 396–410.

[24] Yayeh B, Fekremariam A. Rice (Oryza Sativa) and Chickpea (Cicer aritinum L) Relay Intercropping Systems in an Additive Series Experiment in Rain Fed Lowland Ecosystem of Fogera Vertisols. Journal of Biology, Agriculture and Healthcare. 2014; 4 (27):199–204.

[25] Duivenboodew Van N, Paln M, Studer C, Bielders CL and Beukes DJ. Cropping systems and crop complementarity in dry land agriculture to increase soil water use efficiency: a review. Netherlands Journal of Agricultural Science. 2000; 48: 213–236.

[26] Heitholt JJ, Farr JB Eason R. Plant configuration and cultivar environments. Crop Sci. 2005; 45:1800–1808.

[27] Nthabiseng RT, Irvine MK and Moshibudi MP. Response of a maize or dry bean intercrop to maize density and dry bean arrangement under rainfed conditions. IJAAR 2015; 6(6):18–29.

[28] Isaac A., Oyebisi A.K., Kayode O.s and Adeniji S Mojisola A.S. 2020. Effects of spatial arrangement and population density on the growth and yield of sesame (sesamum indicum l.) in a sesame/maize intercrop. Journal of Agricultural Sciences. 65 (4), 337–350.

[29] Nwaogu E N., Udealor A. and Onyemuwa I.I Effect of Soybean Population and Spatial Arrangement on Nutrient Uptake and Production of ginger/Soybean Intercrop in South-Eastern Nigeria. Nigeria Agricultural Journal. 41(1), 83–90.

[30] Alemayehu A, Tamado T, Nigusie D, Yigzaw D, Kinde T and Wortmann CS. Maize-common bean-lupine intercrops productivity and profitability in maize-based cropping system of Northwestern Ethiopia. Ethiopian Journal of Science and Technology. 2016; 9(2): 69–85.

[31] Minale L, Tilahun T, and Alemayehu A. Determination of nitrogen and phosphorus fertilizer levels in different maize-faba bean intercropping patterns in northwestern Ethiopia. Seventh Eastern and Southern Africa Regional Maize Conference. 2001; pp. 513–518.

[32] Bayu W, Addisu M, Tadesse B and Admassu L. Intercropping tef and sunflower in semi-arid areas of Welo,Ethiopia. Tropical Science. 2007; 47: 16–24.

[33] Yayeh B, Fetien A, Tadess D. Competition Indices of Intercropped Lupine (Lal) and Small Cereals in Additive Series in West Gojam, North Western Ethiopia. American Journal of Plant Sciences. 2014; 5: 1296–1305.

[34] Mihretie F, Tsunekawa A, Bitew Y. Teff [Eragrostis tef (Zucc.)] rain-fed yield response to planting method, seeding density, and row spacing. Agronomy Journal. 2020; 1–12.

[35] Yayeh B, Fikire-Mariam A. Determination of seed rate and inter row spacing for finger millet production (eleusine coracana gaertn.) in north western Ethiopia. Int J Res Rev. 2014b; 1(4):1–7.

[36] Ethiopian Meteorology Agency. Western Amhara Metrological service center, Bahir, Ethiopia, 2016.

[37] Beets WC. Multiple cropping and tropical farming systems. Gower, London, Britain, and West Views Press, Colorado, U.S.A. 1982.

[38] Ofori F and Stern WR. Cereal and legume intercropping systems. Advanced Agronomy. 1987; 41:41–90.

[39] Mead R and Willey RW. The concept of land equivalent ratio and advantages in yields from intercropping. Experimental Agriculture. 1980; 16: 217–228.

[40] EJigu E, Natol B, Tolessa T, Yonas S. determination of appropriate maize common bean arrangement in moisture stress areas of borana, southern Ethiopia. Journal of Ecobiotechnology. 2017; 9: 18–23.

[41] Panda SC. Agronomy. Jedhpur, India: Agrobios (India), 2010.

[42] Hirpa T. Effect of Inter-cropping Row Arrangement on Maize and Common Bean Productivity and the Residual Soil. Global J. Sci. Front. Res. 2014; 14: 289–293

[43] FAO (Food and Agricultural Organization). Food and Agriculture Organization. 2015. Available at www.fao.Org.

[44] SAS Institute Inc. JMP® 13 Basic Analyses. Cary, NC: SAS Institute Inc. 2016.

[45] Gomez K.A. and Gomez A.A. *Statistical procedures for agricultural research*. John Wiley and Sons: New York;1984

[46] Smith RG, Menalled FD and Robertson GP. Temporal yield variability under conventional and alternative management systems. Agronomy Journal. 2007; 99: 1629–1634.

[47] Ahmed S., Raza M.A., Yuan X., et al. Optimized planting time and co-growth duration reduce the yield difference between intercropped and sole soybean by enhancing soybean resilience toward size-asymmetric competition. Food Energy Secur. 2020; 9:e226.

[48] Reddy KC and Visser PL. Cowpea intercrops growth and yield as affected by time of planting relative to millet. African Crop Science 1997; 5:351–357.

[49] Carruthers PN. Phenomenal Consciousness,Cambridge: Cambridge Uni-versity Press. 2000.

[50] Adipala E, Karungi J, Bashasha B, Mugisha J, Asekenye CE, Iceduna C, et al. Dissemination and adoption of an integrated pest management package for groundnut production in Eastern Uganda. In: IPM/CRSP annual report. 2002; 2001–2002.

[51] Silva F, Lambe T., Marr WA. Probability and risk of slope failure. Journal of Geotechnical and Geoenvironmental Engineering. 2008; 134(12): 1691–1699.

[52] Addo-Quaye A, Darkwa A, Ocloo G K. Yield and Productivity of Component Crops in a Maize-Soybean Intercropping System as Affected By Time of Planting and Spatial Arrangement. J. Agric. Biol. Sci. 2011; 6(9): 50–57.

[53] Alemayehu D Shumi D, Afeta T. Effect of Variety and Time of Intercropping of Common Bean (Phaseolus vulgaris L.) With Maize (Zea mays L.) on Yield Components and Yields of Associated Crops and Productivity of the System at Mid-Land of Guji, Southern Ethiopia. Adv Crop Sci Tech. 2018; 6, 324.

[54] Akanbi WB and Togun AO. Productivity and influence of maize stover compost on growth and nutrient uptake of Amaranth. Scientia Horticulture 2002; 93: 1–8.

[55] Singh KK and Rathi KS. Dry matter production and productivity as influenced by staggered sowing of mustard intercropped at different row ratios with chickpea. J. Agron. Crop Sci. 2003; 189:169–175.

[56] Gbaraneh L D, Ikpe FN, Larbi A, Wahua AT, and Torunana MA. The influence of lablab (Lablab purpureus) on grain and fodder yield of maize (Zea mays) in a humid forest region of Nigeria. Journal of Applied Science and Environmental Management. 2004; 8 (2):45–50.

[57] Mousa H, Alfirevic Z. Treatment for primary postpartum haemorrhage. Cochrane Database of Systematic Reviews. 2007; 2007(1):1–25. 10.1002/14651858.CD003249.pub2 17253486

[58] Chen C, Westcott M, Neill K, Wichman D and Knox M. Row configuration and nitrogen application for barley-pea intercropping in Montana. Journal of Agronomy. 2004; 96:1730–1738.

[59] Ashenafi NA. Advantages of Maize-Common bean Intercropping over Sole Cropping through Competition Indices at west Badewacho woreda, Hadiya Zone, SNNPR. Academic Research Journal of Agricultural science and research. 2016; 4: 1–8.

[60] Belstie L, Walelign W, Sheleme B. Determinations of Common Bean (Phaseolus vulgaris L.) Planting Density and Spatial Arrangement for Staggered Intercropping with Maize (Zea mays L.) at Wondo Genet, Southern Ethiopia. Acadamic Research Journal of Agricultural science and research. 2016; 4: 297–320.

[61] Dordas CA, Vlachostergios DN and Lithourgidis A S. Growth dynamics and agronomic-economic benefits of pea-oat and pea-barley intercrops. Crop and Pasture Science. 2012; 63: 45–52.

[62] Ghosh PK. Growth, yield, competition and economics of Groundnut/cereal fodder intercropping systems in the semi-arid tropics of India. Field Crops Research. 2004; 88: 227–237.

[63] Bhadoria RB, Chauhan GS, Kushwaha HS and Singh VN. Intercropping of cluster bean and pearl millet. Indian Journal Agronomy. 1992; 17: 416–439.

[64] Dutta HS, Baroova, and Rajkhowa DJ. Feasibility and economic profitability of wheat (Triticum aestivum)-based intercropping system under rain fed conditions. Indian Journal of Agronomy. 1994; 39: 448–450.

[65] Dhima KV, LithourgidisA S. and Dordas C A. Competition indices of common vetch and cereal intercrops in two seeding ratio. Field Crop Research. 2014; 100: 249–256.

[66] Yu Y, Stomph TJ, Makowski D and van der Werf W. Temporal niche differentiation increases the land equivalent ratio of annual intercrops: a meta-analysis. Field Crops Research. 2015; 184: 133–144.

