# Supplementary material for: Response of maize and common bean to spatial and temporal differentiation in maize-common bean intercropping
Source: PLoS One. 2021 Oct 1;16(10):e0257203. doi: 10.1371/journal.pone.0257203 (PMC8486100; doi:10.1371/journal.pone.0257203)
Supplement: S1 Table — (DOCX) [file pone.0257203.s001.docx]

Table S1: Maize row data ready for analysis at Adet

| spatial arrangement | planting time | replication | Plant height in m | Cob length in cm | Stover yield kg/ha | 1000 seed weight (g) | Grain yield (kg/ha) |
| --- | --- | --- | --- | --- | --- | --- | --- |
| 1 | 1 | 1 | 282.4 | 16.2 | 14524.82 | 337.3364 | 11919.62 |
| 1 | 1 | 2 | 275.2 | 16.66 | 16340.43 | 369.9109 | 14485.46 |
| 1 | 1 | 3 | 279.4 | 15.32 | 19063.83 | 387.3864 | 13630.71 |
| 2 | 1 | 1 | 277.2 | 15.42 | 14524.82 | 372.1855 | 12946.21 |
| 2 | 1 | 2 | 295.2 | 15.66 | 17066.67 | 302.5691 | 14146.25 |
| 2 | 1 | 3 | 269 | 16.36 | 16340.43 | 311.2097 | 13791.98 |
| 1 | 2 | 1 | 290.4 | 16.5 | 17248.23 | 384.615 | 13792.63 |
| 1 | 2 | 2 | 282.4 | 15.75 | 16340.43 | 316.1564 | 14485.46 |
| 1 | 2 | 3 | 276.6 | 15.06 | 15432.62 | 354.3545 | 11914.5 |
| 2 | 2 | 1 | 274 | 14.56 | 18156.03 | 352.38 | 12764.75 |
| 2 | 2 | 2 | 285.2 | 15.06 | 17248.23 | 403.7236 | 14481.61 |
| 2 | 2 | 3 | 274.2 | 16.24 | 16703.55 | 321.7645 | 12770.52 |
| 1 | 3 | 1 | 274 | 17 | 12709.22 | 406.5986 | 10553.51 |
| 1 | 3 | 2 | 285.8 | 16.08 | 16340.43 | 353.925 | 12941.73 |
| 1 | 3 | 3 | 274 | 14.66 | 16885.11 | 310.2909 | 15330.91 |
| 2 | 3 | 1 | 282.2 | 14.64 | 17248.23 | 361.88 | 12775.33 |
| 2 | 3 | 2 | 276.6 | 15.94 | 18156.03 | 375.9355 | 13638.08 |
| 2 | 3 | 3 | 281 | 14.52 | 16340.43 | 326.865 | 14476.16 |

Factor 1: common bean planting time

1= simultaneously with maize

2 = at emergence of maize

3 = at knee height of maize

Factor 2: Spatial arrangement

1 = alternate

2 = paired
