# Supplementary material for: Response of maize and common bean to spatial and temporal differentiation in maize-common bean intercropping
Source: PLoS One. 2021 Oct 1;16(10):e0257203. doi: 10.1371/journal.pone.0257203 (PMC8486100; doi:10.1371/journal.pone.0257203)
Supplement: S2 Table — (DOCX) [file pone.0257203.s002.docx]

Table S2: Common bean row data ready for analysis at Adet

| Spatial arrangement | planting date | replication | Plant height in m | Pod per plant | Sees per plant | Biomass yield (kg/ha) | Grain yield (kg/ha) | 1000 Wight (g) |
| --- | --- | --- | --- | --- | --- | --- | --- | --- |
| 1 | 1 | 1 | 93.7 | 19.7 | 6.7 | 4051.064 | 1190.616 | 258.8 |
| 1 | 1 | 2 | 99.1 | 11.8 | 6.1 | 3812.766 | 844.8453 | 249.4 |
| 1 | 1 | 3 | 89.2 | 18.8 | 6.7 | 4051.064 | 935.0809 | 266.2 |
| 2 | 1 | 1 | 86.4 | 23.5 | 7.4 | 4629.787 | 1383.671 | 278.2 |
| 2 | 1 | 2 | 91.7 | 24.9 | 6.5 | 4357.446 | 1816.375 | 243.4 |
| 2 | 1 | 3 | 89.5 | 26.6 | 7.6 | 3506.383 | 1263.773 | 270 |
| 1 | 2 | 1 | 75.2 | 19.7 | 6.3 | 2224.113 | 616.8738 | 259.5 |
| 1 | 2 | 2 | 88 | 22.1 | 5.8 | 2780.141 | 618.6213 | 273 |
| 1 | 2 | 3 | 75.5 | 17.6 | 5.6 | 2065.248 | 603.3702 | 268 |
| 2 | 2 | 1 | 68.5 | 23.7 | 7.4 | 2485.106 | 583.4326 | 250.2 |
| 2 | 2 | 2 | 88.6 | 21.2 | 7.9 | 1770.213 | 708.4596 | 273.6 |
| 2 | 2 | 3 | 75.3 | 21.3 | 5.3 | 2859.575 | 864.7036 | 276.4 |
| 1 | 3 | 1 | 12.7 | 1.8 | 1.7 | 158.8652 | 5.661957 | 72 |
| 1 | 3 | 2 | 19.6 | 1.4 | 1.9 | 238.2979 | 12.97135 | 72.5 |
| 1 | 3 | 3 | 15.6 | 2.7 | 2.5 | 397.1631 | 16.93345 | 71 |
| 2 | 3 | 1 | 11.2 | 3.6 | 1.8 | 204.2553 | 7.886298 | 69.5 |
| 2 | 3 | 2 | 11.1 | 1.6 | 2 | 204.2553 | 19.57787 | 72 |
| 2 | 3 | 3 | 15.1 | 2.3 | 2.2 | 306.383 | 20.91779 | 73 |

Factor 1: common bean planting time

1= simultaneously with maize

2 = at emergence of maize

3 = at knee height of maize

Factor 2: Spatial arrangement

1 = alternate

2 = paired
