# Supplementary material for: Response of maize and common bean to spatial and temporal differentiation in maize-common bean intercropping
Source: PLoS One. 2021 Oct 1;16(10):e0257203. doi: 10.1371/journal.pone.0257203 (PMC8486100; doi:10.1371/journal.pone.0257203)
Supplement: S3 Table — (DOCX) [file pone.0257203.s003.docx]

Table S3: Land equivalent ratio row data ready for analysis at Adet

| combined Treatment | replications | year | Maize partial land equivalent ratio | common ban partial land equivalent ratio | Total land equivalent ratio |
| --- | --- | --- | --- | --- | --- |
| 1 | 1 | 1 | 1.098464 | 0.642289 | 1.740752 |
| 1 | 2 | 1 | 0.880312 | 0.463189 | 1.343501 |
| 1 | 3 | 1 | 0.853997 | 0.502714 | 1.356711 |
| 2 | 1 | 1 | 0.76796 | 0.962374 | 1.730334 |
| 2 | 2 | 1 | 0.953767 | 1.168824 | 2.122592 |
| 2 | 3 | 1 | 1.065289 | 0.744808 | 1.810096 |
| 3 | 1 | 1 | 0.977084 | 0.439103 | 1.416187 |
| 3 | 2 | 1 | 1.123856 | 0.471218 | 1.595074 |
| 3 | 3 | 1 | 0.854596 | 0.462571 | 1.317168 |
| 4 | 1 | 1 | 0.822821 | 0.516919 | 1.33974 |
| 4 | 2 | 1 | 0.731648 | 0.389079 | 1.120726 |
| 4 | 3 | 1 | 1.062705 | 0.750366 | 1.813071 |
| 5 | 1 | 1 | 1.048924 | 0.008804 | 1.057728 |
| 5 | 2 | 1 | 0.953029 | 0.02017 | 0.973199 |
| 5 | 3 | 1 | 0.879251 | 0.026331 | 0.905582 |
| 6 | 1 | 1 | 0.990126 | 0.012263 | 1.002389 |
| 6 | 2 | 1 | 1.100724 | 0.030443 | 1.131168 |
| 6 | 3 | 1 | 0.657039 | 0.032527 | 0.689566 |
| 7 | 1 | 1 | 1 | 0 | 1 |
| 7 | 2 | 1 | 1 | 0 | 1 |
| 7 | 3 | 1 | 1 | 0 | 1 |
| 8 | 1 | 1 | 0 | 1 | 1 |
| 8 | 2 | 1 | 0 | 1 | 1 |
| 8 | 3 | 1 | 0 | 1 | 1 |
| 1 | 1 | 2 | 0.900987 | 0.611176 | 1.512162 |
| 1 | 2 | 2 | 1.094934 | 0.429927 | 1.524861 |
| 1 | 3 | 2 | 1.030325 | 0.480874 | 1.511199 |
| 2 | 1 | 2 | 0.978585 | 0.601124 | 1.579709 |
| 2 | 2 | 2 | 1.069294 | 0.836878 | 1.906172 |
| 2 | 3 | 2 | 1.042515 | 0.616857 | 1.659373 |
| 3 | 1 | 2 | 1.042564 | 0.262914 | 1.305478 |
| 3 | 2 | 2 | 1.094934 | 0.248054 | 1.342988 |
| 3 | 3 | 2 | 0.900599 | 0.240437 | 1.141036 |
| 4 | 1 | 2 | 0.964869 | 0.197294 | 1.162163 |
| 4 | 2 | 2 | 1.094643 | 0.360187 | 1.454831 |
| 4 | 3 | 2 | 0.965305 | 0.300375 | 1.265679 |
| 5 | 1 | 2 | 0.797724 | 0 | 0.797724 |
| 5 | 2 | 2 | 0.978246 | 0 | 0.978246 |
| 5 | 3 | 2 | 1.15884 | 0 | 1.15884 |
| 6 | 1 | 2 | 0.965668 | 0 | 0.965668 |
| 6 | 2 | 2 | 1.030882 | 0 | 1.030882 |
| 6 | 3 | 2 | 1.094232 | 0 | 1.094232 |
| 7 | 1 | 2 | 1 | 0 | 1 |
| 7 | 2 | 2 | 1 | 0 | 1 |
| 7 | 3 | 2 | 1 | 0 | 1 |
| 8 | 1 | 2 | 0 | 1 | 1 |
| 8 | 2 | 2 | 0 | 1 | 1 |
| 8 | 3 | 2 | 0 | 1 | 1 |

Factor 1: common bean planting time

1= simultaneously with maize

2 = at emergence of maize

3 = at knee height of maize

Factor 2: Spatial arrangement

1 = alternate

2 = paired
