# Supplementary material for: Response of maize and common bean to spatial and temporal differentiation in maize-common bean intercropping
Source: PLoS One. 2021 Oct 1;16(10):e0257203. doi: 10.1371/journal.pone.0257203 (PMC8486100; doi:10.1371/journal.pone.0257203)
Supplement: S4 Table — (DOCX) [file pone.0257203.s004.docx]

Table S4: Maize row data ready for analysis at Finotslam

| spatial arrangement | Planting time | replication | Plant height (cm) | Biomass yield kg/ha | 1000seed weight (g) | Grain yield (kg/ha) |
| --- | --- | --- | --- | --- | --- | --- |
| 1 | 1 | 1 | 304.5 | 13241.61 | 335.8545 | 6560.258 |
| 1 | 1 | 2 | 328.5 | 13006.15 | 336.654 | 6777.441 |
| 1 | 1 | 3 | 337.5 | 9121.513 | 319.3898 | 4249.311 |
| 2 | 1 | 1 | 298.5 | 7294.563 | 324.619 | 5215.022 |
| 2 | 1 | 2 | 308 | 11173.52 | 311.3216 | 8035.204 |
| 2 | 1 | 3 | 309.5 | 10944.68 | 304.979 | 7519.507 |
| 1 | 2 | 1 | 327 | 14624.11 | 303.3723 | 8731.398 |
| 1 | 2 | 2 | 324.5 | 14498.35 | 335.7273 | 7676.454 |
| 1 | 2 | 3 | 313 | 14468.09 | 365.4557 | 6630.053 |
| 2 | 2 | 1 | 307.5 | 12157.92 | 361.7759 | 9075.945 |
| 2 | 2 | 2 | 334 | 14055.79 | 354.3182 | 8975.977 |
| 2 | 2 | 3 | 303 | 14708.28 | 325.1989 | 10203.41 |
| 1 | 3 | 1 | 332.5 | 19083.69 | 364.5545 | 10014.05 |
| 1 | 3 | 2 | 293 | 14916.31 | 330.725 | 8576.802 |
| 1 | 3 | 3 | 317 | 14956.03 | 347.9972 | 7414.52 |
| 2 | 3 | 1 | 326.5 | 13384.4 | 349.9855 | 10591.21 |
| 2 | 3 | 2 | 335 | 15475.18 | 325.733 | 10281.37 |
| 2 | 3 | 3 | 322.5 | 11037.35 | 344.4591 | 6647.569 |

Factor 1: common bean planting time

1= simultaneously with maize

2 = at emergence of maize

3 = at knee height of maize

Factor 2: Spatial arrangement

1 = alternate

2 = paired
