# Supplementary material for: Response of maize and common bean to spatial and temporal differentiation in maize-common bean intercropping
Source: PLoS One. 2021 Oct 1;16(10):e0257203. doi: 10.1371/journal.pone.0257203 (PMC8486100; doi:10.1371/journal.pone.0257203)
Supplement: S5 Table — (DOCX) [file pone.0257203.s005.docx]

Table S5: Common bean row data ready for analysis at Finotslam

| Spatial arrangement | Planting time | Replication | Plant height | Pod/plant | Seed/pod | Seed yield (kg/ha) | Biomass yield (kg /ha) | 1000 see weight (g) |
| --- | --- | --- | --- | --- | --- | --- | --- | --- |
| 1 | 1 | 1 | 51.7 | 22.6 | 7.3 | 992.9078 | 2819.858 | 291.1 |
| 1 | 1 | 2 | 39.8 | 18.7 | 7.7 | 811.8015 | 2303.546 | 276.7 |
| 1 | 1 | 3 | 46.7 | 23 | 7.9 | 1253.447 | 3097.872 | 268.75 |
| 2 | 1 | 1 | 61 | 22.3 | 8 | 1053.617 | 2712.057 | 297.6 |
| 2 | 1 | 2 | 55.2 | 18.1 | 7.7 | 751.0922 | 1753.192 | 257.3 |
| 2 | 1 | 3 | 52.9 | 16.2 | 7.8 | 754.0425 | 2990.071 | 271.3 |
| 1 | 2 | 1 | 63.1 | 23.7 | 7.4 | 840.3971 | 2184.397 | 272.25 |
| 1 | 2 | 2 | 55.8 | 17.3 | 7.6 | 409.8724 | 1707.801 | 253.8 |
| 1 | 2 | 3 | 58.1 | 16.7 | 8.4 | 390.0142 | 1588.652 | 283 |
| 2 | 2 | 1 | 55.8 | 19.3 | 7.8 | 677.3333 | 1968.794 | 265.1 |
| 2 | 2 | 2 | 64.3 | 22 | 7.4 | 756.766 | 1980.142 | 284.4 |
| 2 | 2 | 3 | 57.1 | 16.1 | 8 | 572.8226 | 1702.128 | 266.9 |
| 1 | 3 | 1 | 33.2 | 15.2 | 7.1 | 478.1844 | 996.8794 | 249.95 |
| 1 | 3 | 2 | 34.7 | 12.2 | 8 | 362.2127 | 937.3049 | 246.8 |
| 1 | 3 | 3 | 30.5 | 10.2 | 6.4 | 215.2624 | 536.1702 | 214 |
| 2 | 3 | 1 | 35.6 | 12.9 | 8.2 | 313.1915 | 652.2606 | 228.3 |
| 2 | 3 | 2 | 34.3 | 8.8 | 9.2 | 342.1276 | 819.6809 | 231.8 |
| 2 | 3 | 3 | 35.3 | 18.9 | 8.6 | 362.8937 | 958.8653 | 229.95 |

Factor 1: common bean planting time

1= simultaneously with maize

2 = at emergence of maize

3 = at knee height of maize

Factor 2: Spatial arrangement

1 = alternate

2 = paired
