# Supplementary material for: Response of maize and common bean to spatial and temporal differentiation in maize-common bean intercropping
Source: PLoS One. 2021 Oct 1;16(10):e0257203. doi: 10.1371/journal.pone.0257203 (PMC8486100; doi:10.1371/journal.pone.0257203)
Supplement: S6 Table — (DOCX) [file pone.0257203.s006.docx]

Table S6: Land equivalent ratio row data ready for analysis at Finotslam

| Combined treatment | replication | Year | Maize partial land equivalent ratio | common ban partial land equivalent ratio | Total land equivalent ratio |
| --- | --- | --- | --- | --- | --- |
| 1 | 1 | 1 | 1.393347 | 0.570656 | 1.964003 |
| 1 | 2 | 1 | 1.201793 | 0.523039 | 1.724832 |
| 1 | 3 | 1 | 1.023191 | 0.633391 | 1.656582 |
| 2 | 1 | 1 | 0.858006 | 0.537723 | 1.39573 |
| 2 | 2 | 1 | 1.419217 | 0.310325 | 1.729542 |
| 2 | 3 | 1 | 1.89933 | 0.411391 | 2.310721 |
| 3 | 1 | 1 | 1.021498 | 0.516236 | 1.537734 |
| 3 | 2 | 1 | 0.993137 | 0.163261 | 1.156397 |
| 3 | 3 | 1 | 1.474545 | 0.246403 | 1.720947 |
| 4 | 1 | 1 | 0.864283 | 0.391307 | 1.25559 |
| 4 | 2 | 1 | 1.65753 | 0.459332 | 2.116863 |
| 4 | 3 | 1 | 2.097792 | 0.393899 | 2.49169 |
| 5 | 1 | 1 | 1.838424 | 0.390767 | 2.229191 |
| 5 | 2 | 1 | 0.777904 | 0.269078 | 1.046982 |
| 5 | 3 | 1 | 1.154038 | 0.159481 | 1.313519 |
| 6 | 1 | 1 | 1.379465 | 0.18464 | 1.564105 |
| 6 | 2 | 1 | 1.093928 | 0.223512 | 1.31744 |
| 6 | 3 | 1 | 0.543278 | 0.277284 | 0.820562 |
| 7 | 1 | 1 | 1 | 0 | 1 |
| 7 | 2 | 1 | 1 | 0 | 1 |
| 7 | 3 | 1 | 1 | 0 | 1 |
| 8 | 1 | 1 | 0 | 1 | 1 |
| 8 | 2 | 1 | 0 | 1 | 1 |
| 8 | 3 | 1 | 0 | 1 | 1 |
| 1 | 1 | 2 | 0.574497595 | 0.960497178 | 1.534994773 |
| 1 | 2 | 2 | 0.804718624 | 0.873827072 | 1.678545696 |
| 1 | 3 | 2 | 0.689608442 | 1.047169811 | 1.736778254 |
| 2 | 1 | 2 | 0.678505031 | 0.870283019 | 1.54878805 |
| 2 | 2 | 2 | 0.95904034 | 0.757075472 | 1.716115812 |
| 2 | 3 | 2 | 0.81876583 | 0.813701356 | 1.632467186 |
| 3 | 1 | 2 | 1.504810104 | 0.530660377 | 2.035470482 |
| 3 | 2 | 2 | 1.238528049 | 0.424528302 | 1.663056351 |
| 3 | 3 | 2 | 1.371673839 | 0.477620873 | 1.849294712 |
| 4 | 1 | 2 | 0.981176168 | 0.474056604 | 1.455232772 |
| 4 | 2 | 2 | 1.007215156 | 0.488207547 | 1.495422703 |
| 4 | 3 | 2 | 0.955145041 | 0.283018868 | 1.238163909 |
| 5 | 1 | 2 | 1.133295954 | 0.120283019 | 1.253578973 |
| 5 | 2 | 2 | 1.678549296 | 0.141509434 | 1.82005873 |
| 5 | 3 | 2 | 1.023179981 | 0.130940448 | 1.154120429 |
| 6 | 1 | 2 | 1.700593148 | 0.212264151 | 1.912857299 |
| 6 | 2 | 2 | 1.868710715 | 0.191037736 | 2.059748451 |
| 6 | 3 | 2 | 1.353985304 | 0.201666105 | 1.555651409 |
| 7 | 1 | 2 | 1 | 0 | 1 |
| 7 | 2 | 2 | 1 | 0 | 1 |
| 7 | 3 | 2 | 1 | 0 | 1 |
| 8 | 1 | 2 | 0 | 1 | 1 |
| 8 | 2 | 2 | 0 | 1 | 1 |
| 8 | 3 | 2 | 0 | 1 | 1 |

Factor 1: common bean planting time

1= simultaneously with maize

2 = at emergence of maize

3 = at knee height of maize

Factor 2: Spatial arrangement

1 = alternate

2 = paired
